# Toll-like receptor 4/nuclear factor-kappa B pathway is involved in activating microphages by polysaccharides isolated from *Fagopyrum esculentum*

**DOI:** 10.1080/21655979.2019.1682214

**Published:** 2019-10-29

**Authors:** Zhen Chen, Leilei Yu, Xiaoniao Cai, Fangpeng Ye, Peisheng Jin

**Affiliations:** Department of Gastroenterology, Third Affiliated Hospital of Wenzhou Medical University, Rui’an People’s Hospital, Rui’an, China

**Keywords:** Buckwheat polysaccharide fractions, macrophages, toll-like receptor 4, inducible nitric oxide synthase, nitric oxide

## Abstract

Buckwheat polysaccharide fractions (BPFs) isolated from seeds of *Fagopyrum esculentum* have shown extensive immunomodulatory activities including activation of immune system. In this study, the immuno-modulation effects of BPFs on microphages were investigated. The obtained results show that BPFs can activate microphages as indicated by significant increases in the activity of inducible nitric oxide synthase (12.6 ± 1.30 U/mg prot), nuclear factor-kappa B (NF-κB) protein levels, and secretion of nitric oxide (NO) (21.5 ± 1.20 μmol/ml) and tumor necrosis factor-alpha (TNF-α) (71.2 ± 18.20 pg/ml). Moreover, blocking toll-like receptor 4 (TLR4)/NF-κB pathway using a specific antibody to TLR4 or inhibitor of NF-κB led to the significant inhibitory immuno-modulation effect on microphages as indicated by the decrease in the secretion level of NO and TNF-α. It is demonstrated that BPFs can activate microphages and TLR4/NF-κB pathway is involved in the induction of NO and TNF-α in macrophages by BPFs.

## Graphical Abstract

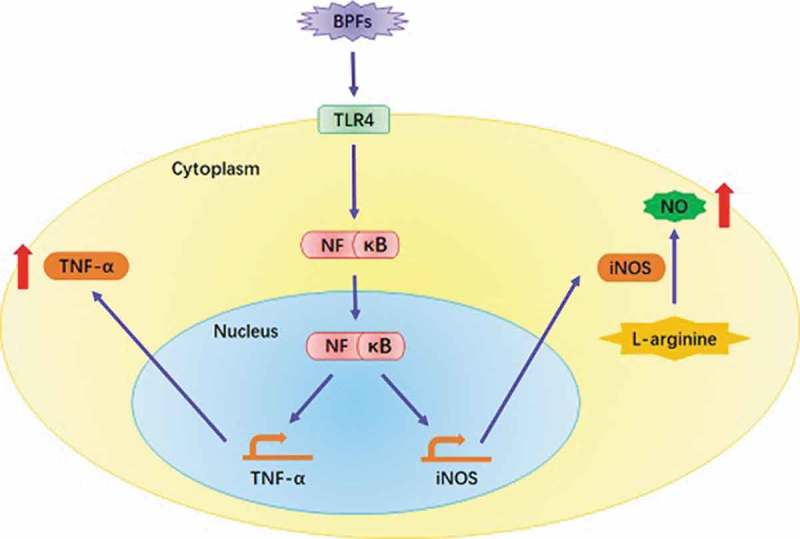

● BPFs were found capable of activating macrophages.

● BPFs increase the NO and TNF-α secretion of macrophages.

## Introduction

Immuno-modulation is to manipulate the immune system to control the infections and other adverse health effects with precise regulation to avoid any complications while suppressive or potentiating efforts are made to benefit the animal and human health [1–3]. It is thus highly desirable and favorable to discover novel substances that have immunomodulatory potentials, especially the substances that show therapeutic effects allowing for the interventions aimed at modifying the immune responses. In recent decades, polysaccharides isolated from plants have attracted more and more attention in biochemical and medical areas due to their extensive biological activities including immuno-modulation and antitumor effects [4–7]. Polysaccharides have great potential to be utilized as novel drugs to overcome the shortcomings of traditional antitumor drugs such as expensive, toxic to normal cells and severe side effects. For instance, buckwheat is one of the most versatile crops and functional foods due to its multiple nutritional compounds that are benefitted to most human health [8,9]. Specifically, buckwheat polysaccharide fractions (BPFs) isolated from the seeds of *Fagopyrum esculentum* have been proved to be an important functional ingredient of buckwheat, which have shown great potential in the activation of immune responses [10,11]. It is reported that BPFs can activate RAW 264.7 macrophage cell line as indicated by the results of MTT assay and measurement on the nitric oxide (NO) production and immune-related cytokine levels [11]. However, the underlying mechanisms of BPFs immuno-modulation activity on macrophages are still unclear. Therefore, the motivation of this study is aim to explore the possible mechanisms of BPFs immuno-modulation activity on macrophages.

Macrophages play very important roles in mammalian immune system, which have been recognized as one of the most important immunocompetent cell types. Macrophages can be activated under a variety of stimuli to serve as killers toward tumor cells and specific pathogens *via* phagocytosis process or secretion of cytotoxic molecules including NO, interleukin-1 (IL-1), tumor necrosis factor-alpha (TNF-α) and reactive oxygen intermediates [12]. It is also indicated that nuclear factor-kappa B (NF-κB) plays critical roles in the secretion of cytokines from macrophages, which is a nuclear transcription factor that regulates expression of a large number of genes. NF-κB migrated to the nucleus binds with the cognate sites in the promoter regions of various genes including many inflammatory cytokine genes to activate their transcription [13]. Previous reports have demonstrated that chitosan is able to stimulate macrophages as indicated by the increase in the secretion of cytokines such as NO and TNF-α [14–16]. However, little is known about the immuno-modulation effects of BPFs on macrophages and the underlying mechanisms.

Therefore, this study investigated the immuno-modulation effects of BPFs on microphages *in vitro* to determine if BPFs can activate the macrophages and the possible signal transduction pathway involved in the activation process. BPFs were used as stimuli and applied to the macrophages *in vitro*. The activity of macrophages was determined by inducible nitric oxide synthase (iNOS), NF-κB protein levels and secretion of NO and TNF-α. To further determine if the toll-like receptor 4 (TLR4)/NF-κB pathway is involved in the activation process of macrophages, specific antibody to TLR4 or inhibitor of NF-κB was used to block this intracellular signal transduction pathway to investigate the role of this pathway in the activation of macrophages.

## Results

### BPFs increase NO production and iNOS activity of macrophages

**Figure 1.**
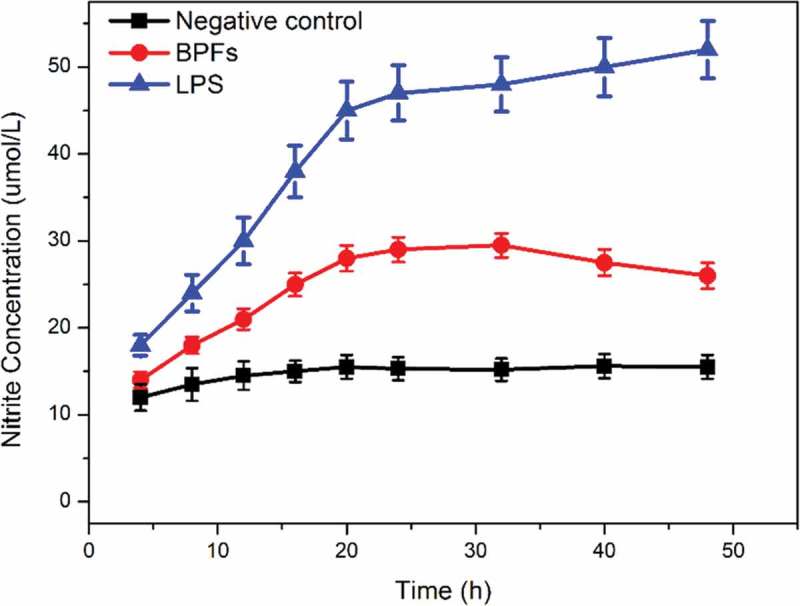
Time-dependent effects of BPFs on NO secretion in RAW264.7 cells. The amount of NO was measured after RAW264.7 cells incubation with medium alone (control), BPFs (10 μg/ml) or LPS (10 μg/ml) for the indicated intervals of time (4, 8, 12, 16, 20, 24, 32, 40, 48 h) by Griess method.

Griess method was employed to measure the amount of NO existed in the culture medium of macrophages for the determination of NO secretion from macrophages under stimulations in a time-dependent manner. BPFs at the concentration of 10 μg/ml were used to stimulate macrophages. Cell culture medium alone and lipopolysaccharide (LPS) (10 μg/ml) were used as the negative and positive controls, respectively. LPS is an amphiphilic glycolipid of the outer membrane of Gram-negative bacteria, which is related to the toxicity and immunogenicity upon infection. Therefore, in this study, LPS was used as a positive control to stimulate macrophages. The results indicate that BPFs significantly increase the secretion of NO from macrophages (Figure 1). The increases in NO production start within 12 h under stimulations of BPFs and continue to accelerate with high rate within 18 h then gradually decrease with lower rate with time until 48 h stimulations. On the contrary, the cell culture medium alone showed no obvious stimulation effects on the NO secretion when applied to macrophages as a negative control. As expected, LPS as the positive control shows much stronger enhancive effects on the NO secretion of macrophages.

**Figure 2.**
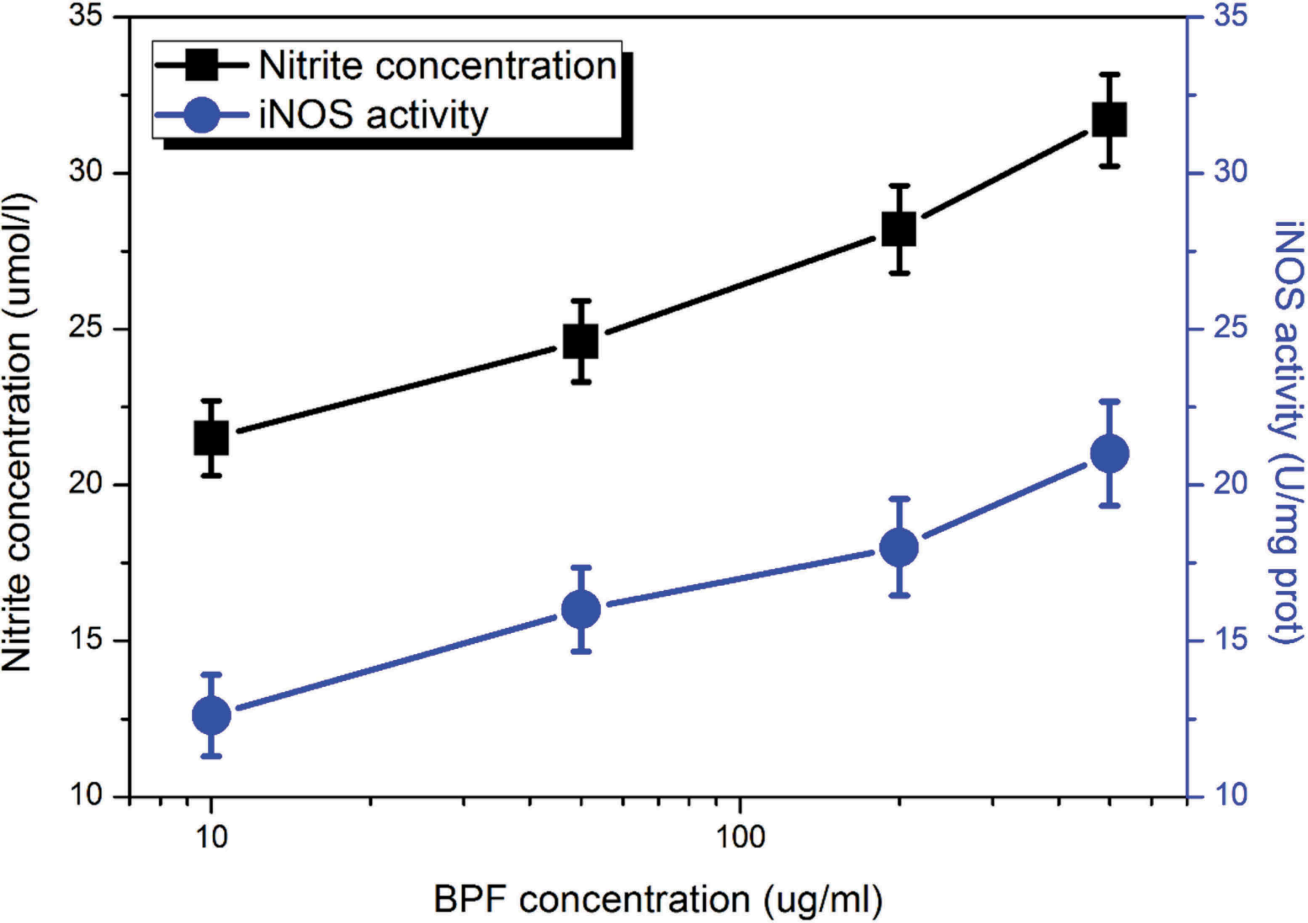
Dose-dependent effects of BPFs on NO production and the activity of iNOS in RAW264.7 cells. All the data were presented as mean ± SD of three independent experiments.

To further determine the NO secretion by macrophages and its relation to the activity of iNOS, different concentrations of BPFs ranging from 10 to 140 μg/ml were applied to stimulate the macrophages for 18 h. The NO secretion and the activity of iNOS of macrophages (adjust to 5 × 10^5^cells/ml) under stimulations of BPFs at different concentrations were measured by Griess method and the iNOS assay kit, respectively. Similarly, the cell culture medium alone and LPS (10 μg/ml) were used as the negative and positive controls, respectively. The results indicate that BPFs induce significant increases in the secretion of NO from macrophages and activities of iNOS (Figure 2). In addition, dose-dependent responses in NO production and iNOS activity of macrophages were obtained. On the other hand, the cell culture medium alone shows negligible effects on the NO production (14.5 ± 1.37 μmol/ml, *p* < 0.01, significantly lower than that of BPFs) and iNOS activity (10.8 ± 1.13 U/mg prot, *p* < 0.01, significantly lower than that of BPFs) of macrophages. LPS shows much significant enhancive effects on the NO production (41.5 ± 4.59 μmol/ml, *p* < 0.01, significantly higher than that of BPFs) and iNOS activity of macrophages (36.8 ± 3.72 U/mg prot, *p*< 0.01, significantly higher than that of BPFs).

### BPFs increase TNF-α secretion and expression of macrophages

**Figure 3.**
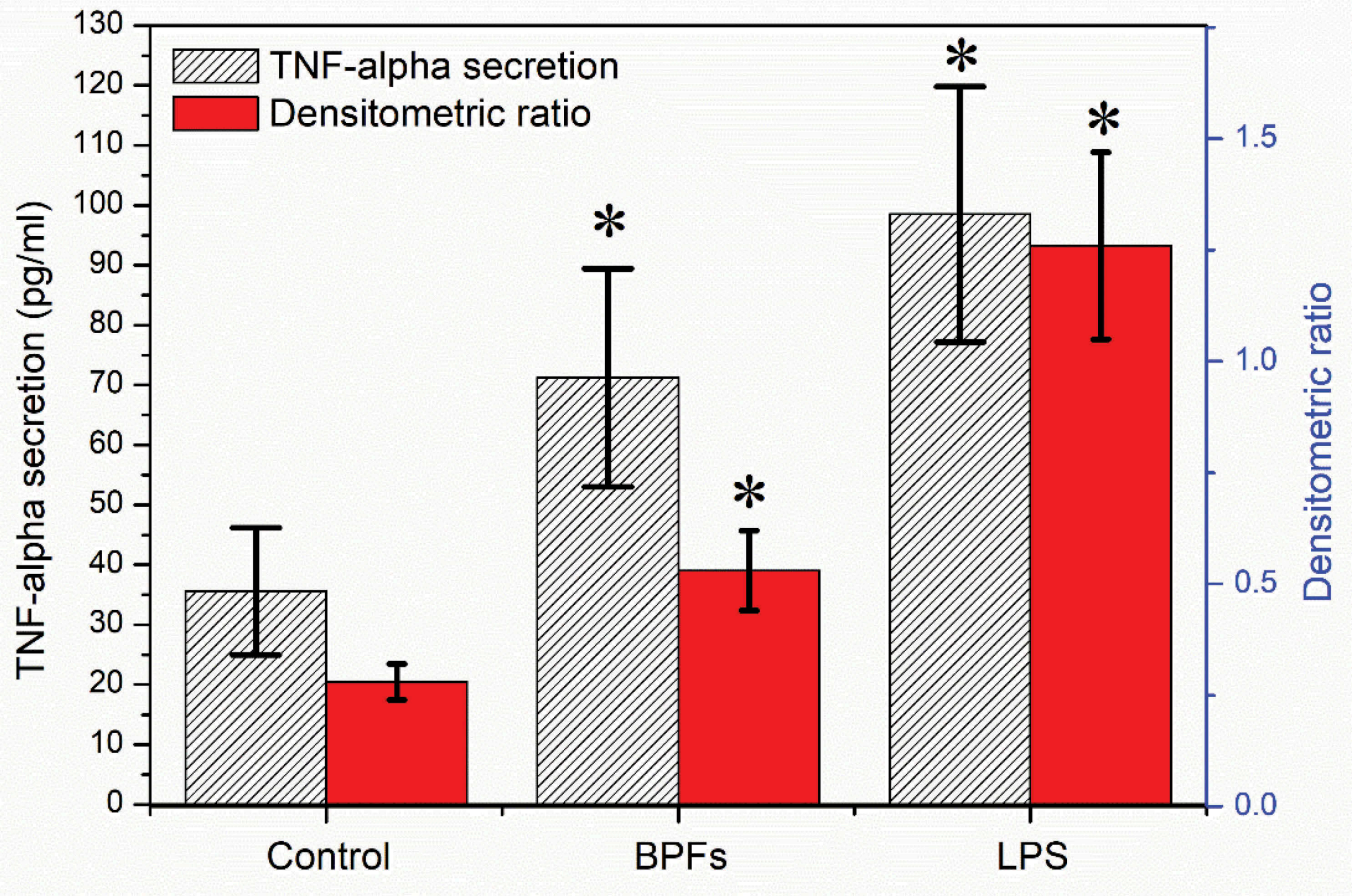
Effects of BPFs on the secretion and expression of TNF-α in RAW264.7 cells. Values were presented as mean ± SD of three independent experiments. *p < 0.01, significantly different to the control.

**Figure 4.**
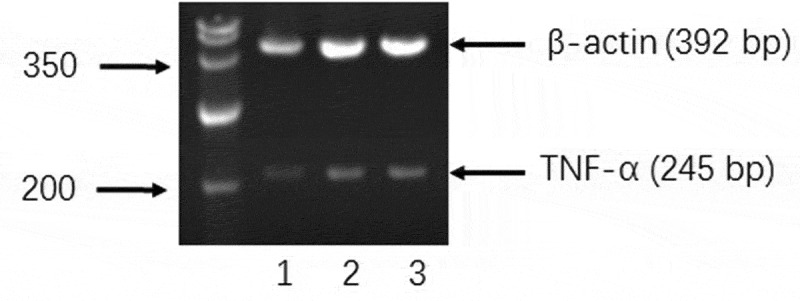
Effects of BPFs on the expression of TNF-α in RAW264.7 cells. RAW264.7 cells were cultured with medium alone (lane 1), BPFs (10 μg/ml) (lane 2), and LPS (10 μg/ml) (lane 3) separately for 18 h.

Enzyme-linked immunosorbent assay (ELISA) and reverse transcription-polymerase chain reaction (RT-PCR) were employed to measure the amount of TNF-α secreted to the cell culture medium by macrophages and the expression of TNF-α at the mRNA level after stimulation of BPFs for 18 h, respectively. The cell culture medium alone and LPS were used as the negative and positive controls, respectively. Macrophages at the concentration of 4 × 10^5^cells/ml after cultured with cell culture medium alone, BPFs (10 μg/ml) and LPS (10 μg/ml) for 18 h were used for ELISA and RT-PCR. The ELISA results show that BPFs stimulation drastically increased the secretion of TNF-α compared to the cell culture medium alone (negative control) (Figure 3). The result of RT-PCR analysis shows the BPFs stimulation significantly increases the transcriptional level of TNF-α compares to the cell culture medium alone (negative control) (Figure 4).

### Effects of NF-κB inhibition on NO and TNF-α secretion

**Figure 5.**
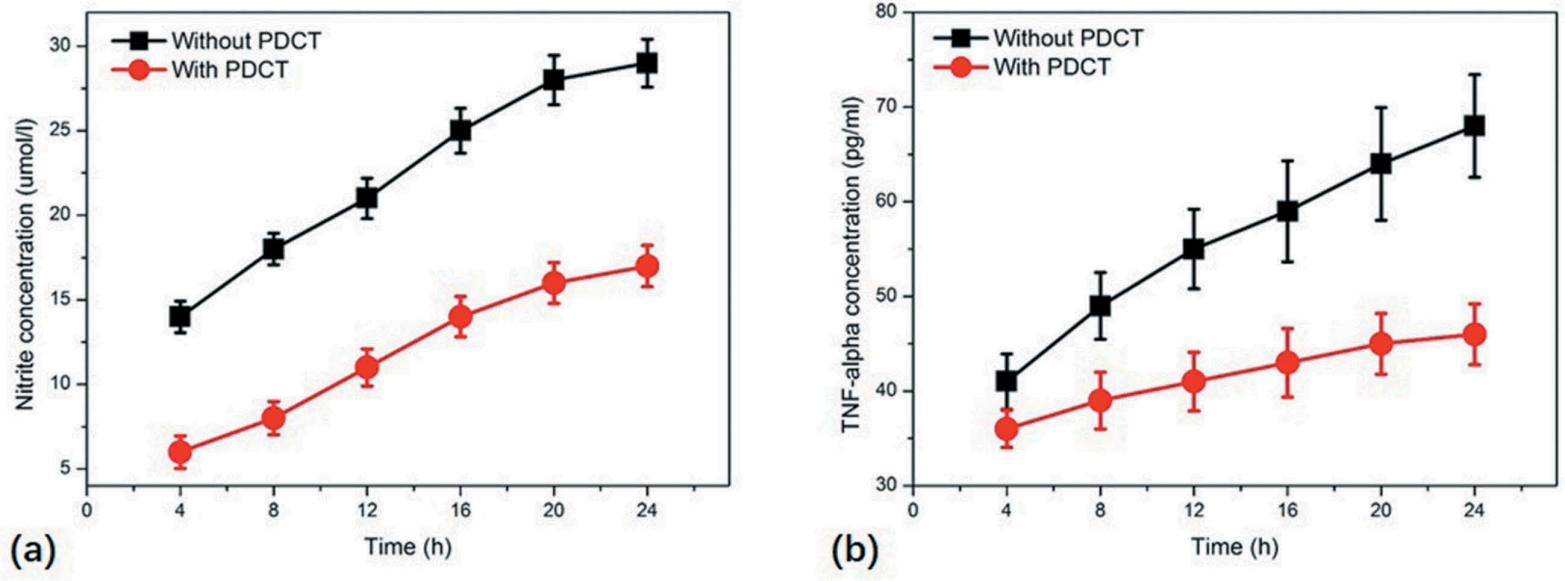
Effects of NF-κB specific inhibitor, pyrrolidinedithiocarbamate (PDTC), on BPF-induced NO (a) and TNF-α (b) production in RAW264.7 cells. RAW264.7 cells of the experimental groups were incubated with PDTC (100 μM) for 90 min, then the cells were stimulated with BPFs (10 μg/ml). The cells of the control groups were incubated with BPFs (10 μg/ml) alone. Aliquots of supernatants were collected to analyze the production of NO and TNF-α at various time intervals (4, 8, 12, 16, 20, 24 h). Values were presented as mean ± SD of three independent experiments.

To investigate the signal transduction mechanisms of NO and TNF-α secretion from macrophages, pyrrolidinedithiocarbamate (PDTC) that is a specific inhibitor of NF-κB was applied to macrophages to explore the potential roles of NF-κB in the production of NO and TNF-α induced by BPFs. Microphages were incubated with PDTC (100 μM) for 90 min and then treated with BPFs (10 μg/ml) for stimulation. The secretion of NO and TNF-α from macrophages was determined using the methods mentioned above. The results indicate that the secretion of both NO and TNF-α from macrophages treated with PDTC was significantly decreased compared to that of macrophages without PDTC treatment (Figure 5).

### NF-κB concentration measurement in nucleus of macrophages

**Figure 6.**
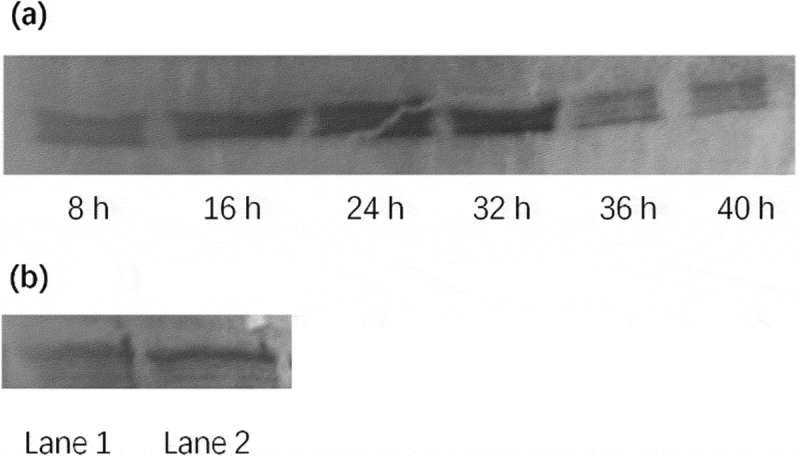
Western blot analysis of nuclear factor-kappaB (NF-κB) in RAW264.7 cells. (a) Time-course changes of the NF-κB content in nuclear extract in RAW264.7 cells treated with BPFs. The RAW264.7 cells were stimulated with BPF (10 μg/ml) for the indicated intervals of time (8, 16, 24, 32, 36, 40 h). (b) Effect of BPFs on the nuclear NF-κB activation in RAW264.7 cells. The RAW264.7 cells of the experimental group (lane 2) were stimulated with BPFs (10 μg/ml) for 6 h and the cells (6 × 10^6^cells/ml) of the control group (lane 1) were incubated with medium alone for 6 h.

To determine the activation of NF-κB, the changes in the concentration of NF-κB in nucleus of macrophages after incubation with BPFs (10 μg/ml) were measured using Western blot analysis. The results indicate that the concentration of NF-κB in nucleus of macrophages with BPFs stimulation increased drastically compared to that of without BPFs stimulation, which increased gradually after 8 h stimulation and reached a peak at 24 h and maintained the peak level until 32 h (Figure 6(a)). In addition, the content of p65 in the nuclear extract from macrophages with BPFs stimulation was significantly higher than that of without BPFs stimulation (Figure 6(b)).

## Discussion

After activation, hundreds of kinds of bioactive molecules are produced by macrophages, which include many bioactive molecules related to immune responses and inflammation such as NO, IL-1, and TNF [17]. In addition, the cytotoxic effect on tumor cells is positively correlated to the yield of NO [18]. As a result, this study investigated the effects of BPFs on the production of NO by mice macrophages. The obtained results showed that BPFs can induce the increase in the secretion of NO from macrophages in a dose- and time-dependent manner. This suggests that activated macrophages by BPFs have immunomodulatory and potential antitumor activities. It is also indicated that the dose–effect relationship between activity increase in iNOS and release rate of NO was consistent. This suggests that BPFs probably increase the secretion of NO via the induction of increase in iNOS activity. Moreover, the increased secretion by promoting synthesis and regulation of macrophage production is probably occurred at transcriptional level of iNOS mRNA.

In macrophages, NO is produced from the catalytic substrate of L-arginine by iNOS. Activity of iNOS depends on the gene transcription but not Ca^2+^/CaM. The action is slow and persistent, which may be activated by LPS or some cell factors such as TNF-α, IFN-γ and IL-1 [12,19–21]. Therefore, this study utilized LPS to stimulate macrophages to serve as the positive control, which can not only objectively evaluate the responses of cells to external stimuli, but also compare to the macrophages in the experimental group stimulated by BPFs. The reagent polymyxin B (PMB) as a specific LPS antagonist can bind to the half group of Lipid A to inhibit the activity of LPS. Since the NO production by macrophages under the stimulation of BPFs was not inhibited by the PMB, the possibility of LPS pollution on BPFs can be eliminated.

iNOS is a non-calcium-dependent type, represented by the iNOS existed in the cytoplasm of inflammatory cells such as white blood cells, which can be induced to respond to inflammation caused by inflammatory cytokines and LPS and catalyze excessive production of NO that can maintain at a high-concentration level for a long time. This phenomenon mainly occurs in the pathophysiological process of the body [22]. NF-κB is a class of proteins that can specifically bind to multiple promoter sites and promote transcription. When cells are stimulated by extracellular signals such as LPS, IκB undergoes phosphorylation degrades and dissociates with NF-κB, which allows for NF-κB quick translocation from cytoplasm to nucleus and specifically binding to the κB site of target genes such as genes encoding iNOS, TNF-α and promoting the transcription of related genes [23,24]. The gene encoding iNOS has a binding site of NF-κB in its regulatory region [25]. In this study, PDTC as an inhibitor significantly inhibited the NO secretion of macrophages induced by BPFs at various time points (8, 16, 24, 32 and 40 h), suggesting that NF-κB signal transduction pathway is involved in the activation of macrophages by BPFs. Since the activation of macrophages by BPFs was not completely blocked, it indicated that other signaling pathways are also involved in the activation of macrophages by BPFs.

Polysaccharides are polymer compounds, which are not able to penetrate the cell membrane and are hardly absorbed in the gastrointestinal tract. However, many studies have shown that polysaccharides are effective by oral administration, intraperitoneal injection and intravenous administration. This suggests that polysaccharides may mediate immune responses through cell membrane receptors. In recent years, there have been exploratory reports about polysaccharide receptors, such as macrophage β-glucan receptor [26], mannose receptor [27], polysaccharide receptor of red blood cells and scavenger receptor. TLR-4 is an important endotoxin LPS receptor, which is an important component of the body’s natural immune system [28]. After TLR-4 activation by LPS, the release of cytokines such as NO is promoted. Recently, many literature have reported that polysaccharides can activate macrophages, T cells, and B cells. Compared with wild-type mouse macrophages, the peritoneal macrophages of the TLR-4 expression-deficient mice were not sensitive to the stimulation of Platycodon grandiflorum polysaccharides, and the yield of NO production induced by stimulations of polysaccharides was very low [29]. In addition, monoclonal antibodies of TLR-4 could block the release of NO induced by Platycodon grandiflorum polysaccharides in wild-type mouse macrophages. Polysaccharides extracted from *Astragalus membranaceus*, and seed cell wall and bark root of *Acanthopanax senticosus*, have been found to activate macrophages via TLR-4 [30,31]. Therefore, TLR-4 of mouse macrophage cell lines was used as candidate receptors for BPFs to explore the roles of BPFs in the activation of mouse macrophages and to reveal the underlying mechanisms. In this study, the results of TLR-4 antibody blocking experiments showed that TLR-4 antibody could block the NO release of macrophages stimulated by BPFs to a certain extent, but its effect was significantly lower than that of TLR-4 antibody to LPS stimulations. This suggests that TLR-4 is the key pathway of LPS activation, but a partial pathway of activation by BPFs, which may be due to many targets and weak effects of polysaccharides *in vivo*, such as TLR-2 of macrophages, β-glucan receptors, mannose receptors, and CR3 receptors. Compared with previous reports [10,11], the key finding of this research work is that TLR-4/NF-kappa B pathway is involved in the activating microphages by BPFs. However, it is still far from the complete understanding of the mechanisms of BPFs immuno-modulation activity. It is thus highly essential to have more studies to investigate if TLR-4 is the main receptors for BPFs in order to reveal the mechanism of BPFs in immune modulation. It is of great significance to explore the roles of other receptors or signaling pathways in the production of NO in macrophages induced by BPFs as well as their synergistic effects for the sake of revealing the immune-regulatory mechanisms of BPFs.

## Materials and methods

### Source of polysaccharide

Buckwheat (*F. esculentum*) was purchased from Changjingzhongye Inc., China. Buckwheat seed was used to extract BPFs as previously reported method, which uses 95% ethanol to precipitate polysaccharide fractions [11]. The total sugar content was 82.1% as indicated by the assays using phenol–sulfuric acid colorimetric method. The average molecular weights of BPFs were estimated to be 23,000 as measured by high-performance gel filtration chromatography. The components of BPFs mainly include glucose, galactose, fucose, arabinose, rhamnose,, and xylose. The endotoxin contamination of BPF was around 70.1 EU/mg as detected by a Pierce Limulus amebocyte lysate Chromogenic Endotoxin Quantitation Kit (Thermo Scientific, Rockford, IL, USA), which is considered to be insignificant for various bioactive products. BPFs dissolved in RPMI 1640 cell culture medium were applied to macrophages as stimuli. RPMI 1640 cell culture medium and LPS (serotype 0111:24; Sigma) was used as negative and positive controls, respectively.

### Culture of macrophages

A murine macrophage-like cell line, RAW264.7, was used as a model of macrophages, which was purchased from Shanghai Institute of Biochemistry and Cell Biology, Chinese Academy of Science. RPMI 1640 medium purchased from Invitrogen GIBCO was used as cell culture medium for the culture of RAW264.7 cells by the addition of 10% heat-inactivated fetal bovine serum, 2 mM L-glutamine, 100 U/ml penicillin-G, and 100 U/ml streptomycin. RAW264.7 cells were cultured in a cell incubator (Thermo Fisher Scientific, USA) at 37°C in a humidified atmosphere containing 5% CO_2_.

### Measurement of nitrite secreted from macrophages

The amount of NO secreted from macrophages was measured by the Griess method as previous report [11]. Briefly, the concentration of nitrite in the culture medium was measured using Griess reagents. For this, 100 μl of the culture supernatants was placed in triplicate in a 96-well plate for 10 min incubation with an equal volume of Griess reagent (1% sulfanilamide, 0.1% N-(1-naphthyl)-ethylendiamine dihydrochloride, 2.5% H_3_PO_4_) at room temperature (RT). A microplate reader (Thermo Fisher Scientific, USA) was utilized to read out the absorbance at 540 nm. Sodium nitrite was used as a standard to determine the concentration of nitrite.

### Detection of intracellular iNOS activity

For the detection of intracellular iNOS activity, RAW264.7 macrophages at the concentration of 3 × 10^6^ were washed with ice-cold PBS (pH 7.2) three times after removal of the cell culture medium. Then, the macrophages were suspended in 1 ml of ice-cold PBS and centrifuged at 1000 × *g* for 5 min. For the next, the macrophages were re-suspended in 50 μl of ice-cold cell lysis buffer (20 mM Tris–HCl pH 7.5, 150 mM NaCl, 1 mM EDTA, 1 mM EGTA, 1 mM glycerol, 1% Triton X-100, 1 mM DTT, 1 μg/ml Leupeptin, 1 mM PMSF). After 20 min incubation on ice, the resulting solution was vortexed for 20 min and centrifuged at 15,000 × *g* for 10 min at 4°C in a centrifuge (Eppendorf, Germany). After the determination of protein concentration in the detergent-soluble fraction, the aliquots were stored at −80°C for further experiments. iNOS assay kit purchased from Nanjing Jiancheng Bioengineering Institute, China was utilized to measure the iNOS activity following the product instruction.

### Measurement of TNF-α secreted from macrophages

The amount of TNF-α secreted from macrophages was measured by ELISA method using TNF-α assay kit purchased from DIACLONE. Briefly, the microphage culture supernatants of 50 μl aliquots were placed into a coated ELISA 96-well plate for 2 h incubation at RT. After washed three times by PBS (pH 7.4), the plate was exposed to biotin-conjugated anti-murine TNF-α antibody for 2 h incubation at RT. Then, the plate was washed three times by PBS and exposed to streptavidin-horse radish peroxidase (HRP) for 30 min incubation at RT. Finally, freshly prepared tetramethylbenzidine with H_2_O_2_ solutions were added to the plate to detect the activity of peroxidase. The absorbance values at 450 nm were readout by a microplate reader (Thermo Fisher Scientific, USA). The concentration of TNF-α was calculated using recombinant murine TNF-α as a standard reference.

### TNF-α expression level measured by RT-PCR

The expression of TNF-α gene in macrophages was measured by RT-PCR at the mRNA level. For this, total RNA was extracted from macrophages using Trizol reagent purchased from Sangon, Shanghai following the product instruction. Then, extracted total RNA of 2 μg was used as the template for the transcribing into cDNA using random hexamers (5 μg) and Mo-AMV reverse transcriptase (200 U) in a 20 μl reaction system. For the next, 2 μg cDNA was amplified by PCR using 0.4 μg each of forward and reverse primer and 2.5 U Taq DNA polymerase (Sangon). PCR was performed on a PCR instrument (Biorad, The Netherlands) under the conditions: 5 min, 94°C pre-incubation, followed by 30 cycles of denaturation (30 s, 94°C), annealing (1 min, 55°C), and extension (1 min, 72°C), followed by a final extension of 10 min at 72°C. PCR products were analyzed by 2% agarose gel electrophoresis. The primers for murine TNF-α were 5ʹ-CCCAA ATGGC CTCCC TCTC-3ʹ (forward) and 5ʹ-CAAATCGGCTGACGGTGTGTCC-3ʹ (reverse). The primers for murine β-actin were 5ʹ-GAGAC CTTCA ACACC CCAGC-3ʹ (forward) and 5ʹ-GAACC GCTCA TTGCC AATAG TGTCC-3ʹ (reverse).

### Determination of NF-κB protein levels by Western blot

For the determination of NF-κB protein levels, RAW264.7 macrophages at the concentration of 6 × 10^6^ were suspended in 1 ml of ice-cold PBS (pH 7.2) after removal of the cell culture medium. Then, the resulting solution was centrifuged at 1000 × *g* for 5 min. For the next, the macrophages were re-suspended in 400 μl of ice-cold hypotonic buffer (10 mM HEPES-KOH pH 7.9, 2 mM MgCl_2_, 0.1 mM EDTA, 10 mM KCl, 1 mM DTT, 1 μg/ml Leupeptin, 1 mM PMSF) for 10 min incubation on ice. For the next, the resulting solution was vortexed and centrifuged at 15,000 × *g* (at 4°C for 30 s). By the following, pelleted nuclear protein was re-suspended in 50 μl of ice-cold saline buffer (50 mM HEPES-KOH pH 7.9, 10% glycerol, 300 mM NaCl, 1.5 mM KCl, 0.1 mM EDTA, 1 mM DTT, 1 μg/ml Leupeptin, 1 mM PMSF) for 20 min incubation on ice and followed by vortex and centrifugation at 15,000 × *g* at 4°C for 10 min. The protein concentration was determined and aliquots were stored at −80°C for further experiments.

SDS-polyacrylamide gel electrophoresis (SDS-PAGE) was applied in the separation of the extracted nuclear proteins. For the first, 30 μg of the nucleoproteins was added with the same volume of 2× SDS-PAGE sample buffer and denatured by boiling for 5 min. After centrifugation at 1000 × *g* for 30 s, 10% SDS-PAGE was performed. Then, a transfer electrophoresis cell (Biorad, The Netherlands) was used to transfer protein to PVDF membrane under 100 V potential for 3 h. PVDF membrane was pretreated with methanol immersion for 5 s, rinsing with double steaming water for 2 min, and balanced by the transfer membrane buffer for 15 min. Separated nuclear proteins were then transferred onto nitrocellulose membrane, which was blocked with 5% skim milk in Tris-Buffered Saline Tween-20 (TBST) for 1 h at RT and incubated with rabbit antimurine NF-κB p65 antibody purchased from Rockland, America for 1 h at RT. For the next, the membrane was washed three times using TBST and incubated with HRP conjugated goat antirabbit-IgG (Rockland) for 1 h. Finally, the membrane was visualized by an enhanced chemiluminescence detection system (Amersham, England).

### Statistical analysis

The obtained experimental data were expressed as means ± standard deviation (SD). The statistical analyses were carried out by performing Student’s *t*-test of the data. *p* < 0.05 was considered as statistically significant difference between two groups.
